# Identifying wildlife reservoirs of neglected taeniid tapeworms: Non-invasive diagnosis of endemic *Taenia serialis* infection in a wild primate population

**DOI:** 10.1371/journal.pntd.0005709

**Published:** 2017-07-13

**Authors:** India Schneider-Crease, Randi H. Griffin, Megan A. Gomery, Pierre Dorny, John C. Noh, Sukwan Handali, Holly M. Chastain, Patricia P. Wilkins, Charles L. Nunn, Noah Snyder-Mackler, Jacinta C. Beehner, Thore J. Bergman

**Affiliations:** 1 Department of Evolutionary Anthropology, Duke University, Durham, North Carolina, United States of America; 2 Department of Anthropology, University of Michigan, Ann Arbor, Michigan, United States of America; 3 Department of Veterinary Medicine, Prince Leopold Institute of Tropical Medicine, Nationalestraat 155, Antwerp, Belgium; 4 Department of Parasitology, Faculty of Veterinary Medicine, Salisburylaan 133, Merelbeke, Belgium; 5 Department of Parasitic Diseases and Malaria, Centers for Disease Control and Prevention, Atlanta, Georgia, United States of America; 6 Duke Global Health Institute, Duke University, Durham, North Carolina, United States of America; 7 Department of Psychology, University of Michigan, Ann Arbor, Michigan, United States of America; 8 Department of Ecology and Evolutionary Biology, University of Michigan, Ann Arbor, Michigan, United States of America; University of Cambridge, UNITED KINGDOM

## Abstract

Despite the global distribution and public health consequences of *Taenia* tapeworms, the life cycles of taeniids infecting wildlife hosts remain largely undescribed. The larval stage of *Taenia serialis* commonly parasitizes rodents and lagomorphs, but has been reported in a wide range of hosts that includes geladas (*Theropithecus gelada*), primates endemic to Ethiopia. Geladas exhibit protuberant larval cysts indicative of advanced *T*. *serialis* infection that are associated with high mortality. However, non-protuberant larvae can develop in deep tissue or the abdominal cavity, leading to underestimates of prevalence based solely on observable cysts. We adapted a non-invasive monoclonal antibody-based enzyme-linked immunosorbent assay (ELISA) to detect circulating *Taenia* spp. antigen in dried gelada urine. Analysis revealed that this assay was highly accurate in detecting *Taenia* antigen, with 98.4% specificity, 98.5% sensitivity, and an area under the curve of 0.99. We used this assay to investigate the prevalence of *T*. *serialis* infection in a wild gelada population, finding that infection is substantially more widespread than the occurrence of visible *T*. *serialis* cysts (16.4% tested positive at least once, while only 6% of the same population exhibited cysts). We examined whether age or sex predicted *T*. *serialis* infection as indicated by external cysts and antigen presence. Contrary to the female-bias observed in many *Taenia*-host systems, we found no significant sex bias in either cyst presence or antigen presence. Age, on the other hand, predicted cyst presence (older individuals were more likely to show cysts) but not antigen presence. We interpret this finding to indicate that *T*. *serialis* may infect individuals early in life but only result in visible disease later in life. This is the first application of an antigen ELISA to the study of larval *Taenia* infection in wildlife, opening the doors to the identification and description of infection dynamics in reservoir populations.

## Author summary

Although tapeworm parasites of the genus *Taenia* are globally distributed and inflict enormous socioeconomic and health costs on their hosts, which include humans, little is known about taeniid tapeworms that infect wildlife. This gap in knowledge prevents an assessment of the potential for these parasites to infect humans and production animals and is largely due to the difficulty of conducting standard diagnostic tests on wildlife. To address this gap, we adapted a standard diagnostic assay to be used with dried urine samples. We used urine from geladas, primates endemic to Ethiopia, which are frequently infected with the larval stage of a taeniid tapeworm and exhibit protuberant cysts during advanced infection. The use of this diagnostic test in a wild gelada population allowed us to detect that individuals can be infected without exhibiting observable cysts, and that some individuals may control infection in its early stages. This tool provides information about how a neglected tapeworm functions in a wildlife system and opens the door to the non-invasive identification of tapeworm reservoir hosts that may threaten humans.

## Introduction

Tapeworm parasites of the genus *Taenia* are globally distributed in numerous mammalian hosts, frequently exploiting predator-prey relationships and posing considerable risk to humans. Although the life cycles and zoonotic potential of some taeniids are among the most well known of all tapeworms, due to their importance in human health and evolution [1], the descriptions of other taeniids have been neglected. Particularly enigmatic is *Taenia serialis*, conventionally thought to infect dogs in its adult stage and rodents and lagomorphs in its intermediate stage [2]. Over the past century, extensive taxonomic and morphological confusion and disagreement [1,3] have made it difficult to identify the geographic and phylogenetic distribution of this parasite. Thus, we begin by providing what is, to our knowledge, the first thorough review of *T*. *serialis* biology and zoonotic potential by synthesizing previous case reports. We then describe the antigen enzyme-linked immunosorbent assay (ELISA) that we validated for use with gelada urine samples. Finally, we demonstrate the application of this assay in a free-living population of Ethiopian geladas (*Theropithecus gelada*), the only known primate host of the larval stage of *T*. *serialis*, and provide recommendations for future implementation of this assay in wildlife systems.

### Review: Diversity and zoonotic potential of *T*. *serialis*

Singular among cyclophyllidean tapeworms, taeniid species parasitize mammals in both their adult and larval stages [1]. Taeniid adult stages infect humans and carnivorous species that include canids, felids, hyaenids, mustelids, and viverrids [1, 3] and cause few severe symptoms in healthy hosts [2, 4, 5]. By contrast, taeniid larval stages (metacestodes) generally infect herbivorous artiodactyl, rodent, and lagomorph species [1, 3] and regularly cause extensive muscular and visceral damage [2, 4, 6, 7]. Intermediate hosts become infected when they ingest eggs shed by adult tapeworms harbored in the definitive host, and definitive hosts become infected when, via predation or scavenging, they ingest larvae in infected intermediate hosts [1, 3].

The scientific study of *T*. *serialis* is marked by a tendency to make species-level designations that may not be warranted and, consequently, to underestimate the range of hosts that *T*. *serialis* infects. The *T*. *serialis* metacestode is a thin-walled, translucent structure (coenurus) containing multiple protoscolices, the precursor to the mature scolex that constitutes the attachment end of the adult tapeworm in the definitive host [6]. This metacestode morphology is indistinguishable from that of *T*. *multiceps*, a zoonotic parasite found primarily in sheep [2]. Before the relatively recent emergence of molecular tools [8–13], cases of coenurosis were ascribed to either *T*. *serialis* or *T*. *multiceps* based on now-outdated morphological cues [2, 14, 15] or on infection site predilection (e.g., central nervous system or subcutaneous tissue) [2, 16, 17]. Furthermore, some researchers employed synonyms for *T*. *serialis* (e.g., *T*. *brauni*, *T*. *glomeratus*) based on geographic location or occurrence in a non-rodent or lagomorph host [17, 18].

In addition to taxonomic confusion surrounding metacestode identification, the occurrence of coenurosis ascribed to *T*. *serialis* in non-rodent or lagomorph hosts has been largely overlooked. Although parasitological texts invariably refer to *T*. *serialis* as a parasite of rodents and lagomorphs in its larval stage, it has been reported in a wide range of phylogenetically and geographically diverse hosts. Case studies have described *T*. *serialis* coenurosis in three rodent species [14, 19–22], domestic cats [23–29], two marsupial species [30, 31], two lagomorph species [32–37], and two nonhuman primate species (the greater spot-nosed guenon (*Cercopithecus nictitans*) [38], and the gelada (*Theropithecus gelada*) [39–44]. To our knowledge, only two studies of naturally occurring *T*. *serialis* coenurosis have used molecular tools for species identification [42, 43]. Given the lack of confirmed *T*. *serialis* diagnoses in the literature, including cases in ‘standard’ rodent and lagomorph hosts, it stands to reason that *T*. *serialis* may be more widespread and flexible in its selection of intermediate hosts than previously described.

The historic difficulty of definitively diagnosing *T*. *serialis* coenurosis may have also led to an underestimation of its zoonotic potential. Coenurosis has been recorded in humans across the globe [45, 46], including in Europe [47–59], Africa [60–65], the Middle East [66, 67], and the Americas [68, 69]. Certain authors declined to assign a species [17, 65], while the others ascribed infection to *T*. *serialis* or *T*. *multiceps* based on morphological analysis. Only one study used molecular tools, identifying *T*. *serialis* coenurosis in a man in Nigeria [46]. In sum, the taxonomic uncertainty of coenurosis occurring in animals, including humans, has led to a fragmented record of the global occurrence and distribution of *T*. *serialis* and a potential underestimation of its zoonotic potential and importance to public health.

### An antigen ELISA to investigate larval *T*. *serialis* in wildlife

As humans come into increasing contact with wildlife, understanding the biology and zoonotic potential of *T*. *serialis* is crucial to preventing its transmission to humans and domestic animals. Little is known about the natural dynamics of *Taenia* spp. in wildlife hosts, largely because of the impracticality of obtaining and storing biological samples or performing medical imaging in remote settings and on wildlife. To obtain a more accurate assessment of the prevalence of larval *T*. *serialis* infection in wildlife host species, we adapted an existing monoclonal antibody-based sandwich enzyme-linked immunosorbent assay (ELISA) for the detection of *Taenia* antigen in dried urine samples [70–73]. The monoclonal antibodies (B158C11 and B60H8) used in this assay are specific to the *Taenia* genus, which permits its use in the detection of larval infections of all taeniid species. Indeed, this assay has been used as an epidemiological tool, often complementary to other diagnostic methods, in studies of porcine, bovine, and human cysticercosis [70, 71, 74–78]. Because this assay detects circulating metacestode (larval) antigens, it identifies active infections rather than past exposure identified by antibody assays [75, 77].

Despite the success of this assay in studies of cysticercosis in livestock, the difficulty of obtaining blood or serum samples from humans limited its use in human populations [77, 78]. Thus, two teams [77, 78] adapted the monoclonal antigen test to non-invasively diagnose these diseases in urine. However, the existing protocols for *Taenia* antigen detection in urine are still impractical for implementation in wildlife studies because they require that urine samples be stored at -20°C until processing [77, 78]. Because many wildlife studies are carried out in areas where electricity is absent or inconsistent, the need for refrigeration limits the practicality of these tests in remote areas. We therefore validated the use of dried urine with a modified protocol to investigate sylvatic cycles of *Taenia* transmission.

### Antigen ELISA implementation in geladas (*Theropithecus gelada*)

**Fig 1:**
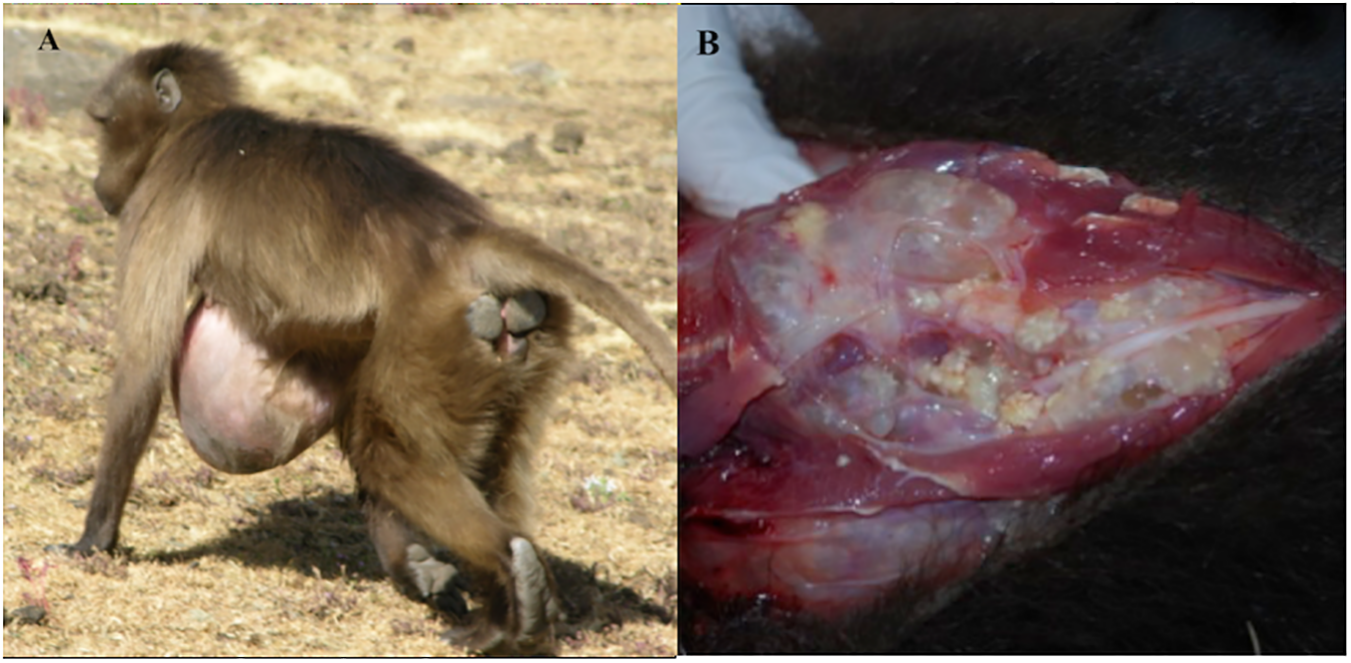
(A) Gelada with a cyst characteristic of confirmed larval *T*. *serialis* infections protruding from the abdomen. (B) Internal view of coenuri in the cyst of an infected individual necropsied upon natural death.

Geladas—herbivorous primates endemic to the Ethiopian highlands–are known to exhibit protuberant cysts characteristic of infection with the larval stage of *T*. *serialis* (Fig 1). Coenuri have been recorded in wild-caught captive geladas for nearly a century and were often ascribed to *T*. *serialis* based primarily on morphological cues [39–41, 79–83]. Recently, this identification was confirmed with molecular diagnosis of cystic material obtained from protuberant cysts [42, 43]. Prevalence of *T*. *serialis*-associated cysts in geladas ranges from 4–13% in an ecologically disturbed area [42, 44, 84] to 30% in an ecologically intact area [43], and cysts in both areas are associated with significant increases in mortality and decreases in reproductive success [43, 44]. However, not all infections necessarily manifest as conspicuous cysts, a point illustrated by the presence of non-protruding cysts revealed during necropsies on infected captive geladas. Thus, prevalence of *T*. *serialis* in geladas based on protuberant cysts is likely to be underestimated.

We implemented the monoclonal antibody-based sandwich ELISA in a wild population of geladas in the Simien Mountains National Park (SMNP), Ethiopia, where individuals are parasitized with *T*. *serialis* [42]. Recent work in this population demonstrated sex- and age- biased distribution of *T*. *serialis* cysts, with higher prevalence in adults and females [44]. This sex bias may reflect either patterns of data collection that bias towards observing infected females and uninfected males, or the estrogen affinity exhibited by the larvae of many taeniid species [85, 86]. The increased prevalence of *T*. *serialis* cysts in adults compared to immatures may arise either from increased susceptibility of adults due to the immunosuppressive effects of hormones related to sexual maturity, or as a function of the time required for infection to develop into observable cysts. The adaptation of the urine antigen ELISA to non-invasively diagnose *T*. *serialis* in dried gelada urine allowed us to investigate infection dynamics that cannot be detected solely by analyzing the presence of observable cysts.

## Materials and methods

### Study site

We conducted our study in the Sankaber area of the SMNP, Amhara Region, Ethiopia. The SMNP was established in 1969 and has been classified as a UNESCO World Heritage Site in Danger since 1996 due to substantial anthropogenic impact [87]. The park covers 13,600 hectares, is characterized by Afro-montane and Afro-alpine habitats, and contains a number of mammals of potential importance to the *T*. *serialis* life cycle. These include the black-backed jackal (*Canis mesomelas*), the golden jackal (*Canis aureus*), the spotted hyena (*Crocuta crocuta*), the Ethiopian wolf (*Canis simiensis*), Starck’s hare (*Lepus starcki*), and the gelada [88]. The substantial human population in the SMNP has contributed to the loss of natural vegetation and the expansion of crops and grazing seen in many areas of the park [88, pers. obs.]. Dogs, jackals, hyenas, and Ethiopian wolves are among the carnivores living in the SMNP that potentially prey on or scavenge the corpses of geladas [88], and are thus of potential importance for the *T*. *serialis* life cycle as definitive hosts.

### Urine sample collection

From August 2014 to June 2015, we collected a total of 527 urine samples from 204 geladas (117 females, 87 males; 37 infants, 60 subadults, 107 adults) in 2 habituated groups under long-term study by the Simien Mountains Gelada Research Project (SMGRP) in the SMNP. Geladas in the habituated groups are each assigned a three-letter code and are individually identifiable by the field team based on suites of morphological characteristics and corporeal idiosyncrasies [88]. Thus, all samples collected in this population were from known individuals, with most individuals sampled more than once over time (n = 97 individuals; median: 2 samples/individual, range: 1–10). Sampling included 58 samples from 10 individuals exhibiting the cysts characteristic of *T*. *serialis* infection to serve as ‘true positives’, and 57 samples from 37 unweaned infants to serve as ‘true negatives’ (unweaned infants are unlikely to ingest eggs because they do not yet eat grass; see below for further explanation). All other samples (412 from 158 individuals) were collected for evaluation in the Ag-ELISA as samples of ‘unknown status’ (median = 2, range: 1–10). These included 94 females and 64 males; 60 subadults and 98 adults.

Urine samples were collected from the ground immediately after urination using Whatman Qualitative Filter Papers (Grade 4, 11.0 cm). After urination, as much urine as possible was soaked up from the ground with a filter paper. The filter paper was folded and stored in a 2-oz Whirl-Pak bag, which was labeled with the unique code associated with the individual, date, and time. Approximately 1 g of indicating silica desiccant was added to each bag to ensure samples remained dry and to prevent mold growth.

### Urine sample analysis

Samples were processed and analyzed using the B158/B60 ELISA (Institute for Tropical Medicine, Antwerp) in the Immunochemistry Laboratory of the Division of Parasitic Diseases and Malaria at the Centers for Disease Control and Prevention (CDC) in Atlanta, Georgia. To aid in identifying urine stains on the filter papers, we viewed each paper under a UV light (long-wave, 365 nm; Spectroline Model ENF-240c), and used an office hole puncher to remove four circles (~6 mm diameter) from the part of each filter paper that was soaked on both sides. The hole puncher was sterilized and dried after each use to prevent cross-contamination. The four circles taken from each sample were placed into a single labeled 2 mL sample tube. Each sample was reconstituted with 1 mL blocking buffer (PBS-Tween 20 + 1% newborn calf serum (NBCS), existing CDC collection) and vortexed.

Following [73], polystyrene ELISA plates (Nunc Maxisorp flat-bottom 96 well) were coated and incubated with the capture antibody (B158C11A1 monoclonal antibody in a sensitization buffer (carbonate bicarbonate buffer, pH 9.5)). Each plate included 80 unknown samples, 4 known negative human samples, and 2 positive control samples created by spiking known negative human samples (existing CDC collection) with 0.125 μg antigen/1 mL urine *T*. *crassiceps* antigen (soluble protein extract). A standard curve (2-fold serial dilutions of known negative human urine samples spiked with *T*. *crassiceps* antigen) was included on each plate as an additional control. After a washing step (1x), plates were coated and incubated with 150 μL/well of blocking buffer, and then loaded and incubated with 100 μL from each sample. After a washing step (4x), plates were coated and incubated with 100 μL of detecting antibody dilution (B60H8A4 + blocking buffer). Plates were washed (1x) and subsequently loaded and incubated with 100 μL of Streptavidin-horseradish peroxidase (HRP) dilution (Peroxidase-conjugated Streptavidin 1:10,000 dilution, Jackson ImmunoResearch Laboratories, West Grove, PA, in blocking buffer (0.1ug/ml)). Plates were washed (1x) and then loaded with 100 μL of Tetramethylbenzidine (TMB) (1-step Ultra TMB-ELISA, ThermoFisher Scientific, USA) and shaken at room temperature for two minutes. After the addition of 100 μL of stop solution (1M sulfuric acid; H_2_SO_4_, EMD Millipore, Darmstadt, Germany) to each well, the optical densities (OD) of samples were read in the VersaMax ELISA Microplate Reader (Molecular Devices, Sunnyvale, CA, USA) at 450 nm (see S1 Text for detailed protocol). If more than one control on a plate failed, the entire plate was repeated. The index value (IV) for each sample relative to the positive and negative controls on each plate was calculated using the following formula:

IV=SampleOD−Average(NegativeControlsOD)/Average(PositiveControlsOD−Average(NegativeControlsOD)

### Receiver operating characteristic analysis

We assessed the sensitivity and specificity of the Ag-ELISA with a receiver operating characteristic (ROC) curve [89]. The nature of working in a wild system precludes establishing a negative ‘gold standard’ because we are unable to confirm negative diagnoses with serological or imaging techniques. Thus, we used unweaned infants as ‘true negatives’ (n = 58 samples), because they do not yet consume grass and are thus minimally exposed to *T*. *serialis* eggs and can be considered likely to be negative. We used individuals presenting with *T*. *serialis* cysts as ‘true positives’ (n = 58 samples). We selected the point on the ROC curve at the shortest distance from the coordinate (0, 1) as the optimal threshold IV for classifying a sample as positive or negative. ROC analysis was performed with the package “pROC” [90] in R [91].

### Analysis of *T*. *serialis* infection predictors in urine samples

To investigate if sex and age predicted the occurrence of cysts among adults and subadults (n = 158 individuals), we used logistic regression implemented in the ‘glm’ function in the R package ‘stats’ [91]. We coded age as a continuous variable based on known or estimated birthdates for individuals. Model selection was performed with Akaike information criterion (AICc), which selects the optimal model based on maximum likelihood [92] with a finite sample size [93].

To investigate if sex and age predicted the occurrence of antigen-positive samples (i.e., those with an IV greater than the IV threshold from the ROC analysis) among adults and subadults without cysts (n = 412 samples, 158 individuals), we used a generalized linear mixed effects model (GLMM) implemented with the ‘glmer’ function in the ‘lme4’ package in R [94]. We used binomial errors with a logit link function, and included age and sex as fixed effects. Because individuals were sampled at varying intensities and may have had different individual risks of infection, we included individual identity as a random effect. We coded age in the following two ways: (1) as a continuous variable based on known and estimated birthdates; and (2) as an ordered categorical variable with two levels based on developmental stage (i.e., subadult or adult). Continuous age is expected to be a relevant predictor of infection if accumulated exposure to *T*. *serialis* eggs in the environment drives risk, whereas categorical age based on developmental stages may be more relevant if hormonal factors are a major driver of risk. We compared the fit of the continuous age and categorical age models using AICc and calculated averaged coefficients for each variable using model averaging.

### Ethics statement

All research was approved by the University Committee on the Use and Care of Animals at the University of Michigan (UCUCA protocol #09554), the Duke University Institutional Animal Care and Use Committee (IACUC protocol #A218-13-08), and followed all laws and guidelines in Ethiopia. This research adhered to the standards presented in the Guide for the Care and Use of Laboratory Animals (National Research Council of the National Academies, 8^th^ Edition) and the Animal Care Policy Manual (United States Department of Agriculture, 2016).

## Results

### Sample analysis

**Fig 2:**
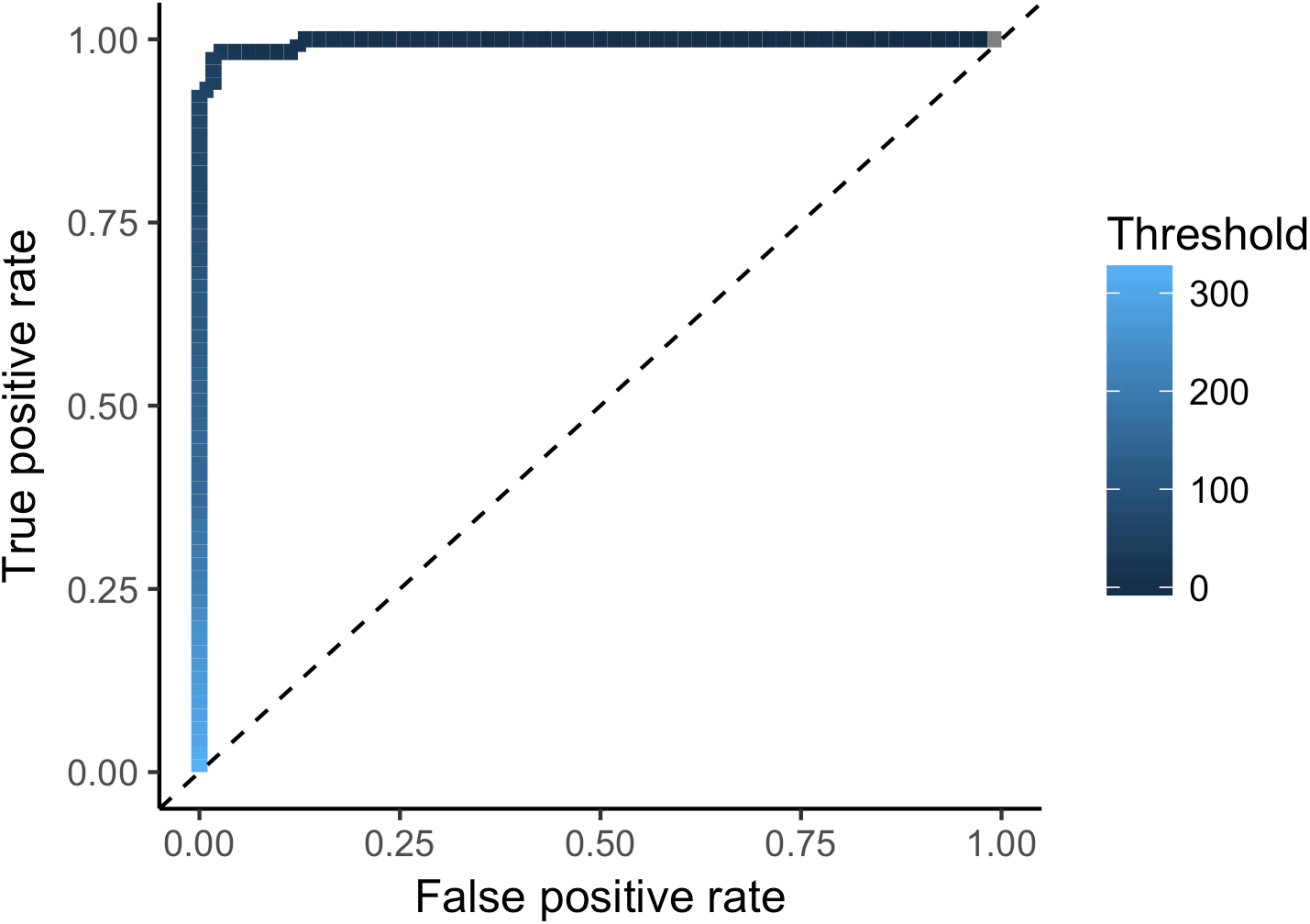
Receiver operator characteristic (ROC) curve of antigen ELISA detection of *T*. *serialis* infection in dried gelada urine. The optimal threshold cutoff index value (42.1) had an estimated specificity of 98.4% (95% CI: 95.1–1) and an estimated sensitivity of 98.5% (95% CI: 95.6–1).

Our measurement of infection status using the described Ag-ELISA was highly accurate. The ROC analysis revealed the optimal threshold IV to be 42.1, with 98.4% specificity (95% CI: 95.1–1), 98.5% sensitivity (95% CI: 95.6–1) and an area under the curve (AUC) of 0.99 (95% CI: 0.9937–1; Fig 2). We identified only one likely false positive (i.e., an infant with a positive sample) (98.2%, 56/57), and one false negative (i.e., an individual with a cyst and a negative sample) (98.3%, 57/58, Table 1). Because sample quality was difficult to evaluate with our collection technique, we binned samples into either ‘positive’ or ‘negative’ categories based on the IV cutoff instead of conducting analysis at the level of sample OD. This conservative approach permits for the broad designation of samples as positive or negative for antigen presence, but precludes analyses that address fluctuations or activity in sample OD.

**Table 1:** Ag-ELISA results of gelada samples (true positive, true negative, unknown status).

	True Positives	True Negatives	Unknown Status
Ag-ELISA: Positive	57 (98.3%)	1 (1.8%)	50 (12.1%)
Ag-ELISA: Negative	1 (1.7%)	56 (98.2%)	362 (87.9%)
Total sample #	58	57	412

True positives = samples from individuals with cysts (n = 10 individuals). True negatives = samples from unweaned infants (n = 37 individuals). Unknown status = samples from individuals without cysts, excluding infants (n = 158 individuals). Positive/negative assigned based on antigen presence above the optimal threshold IV cutoff (an indexed optimal density of 42.1) determined by the ROC analysis.

**Fig 3:**
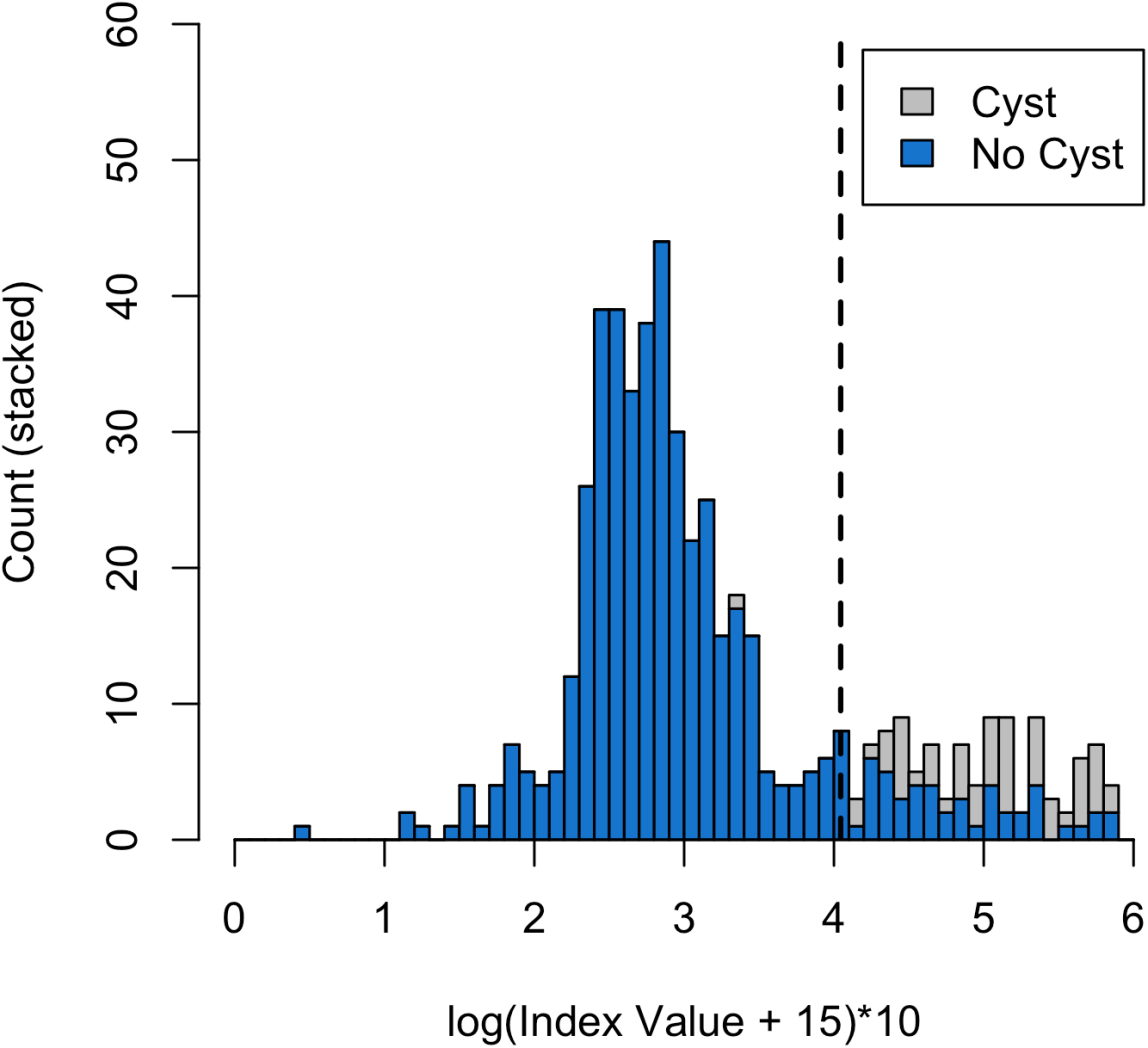
Counts of log sample index values (IVs) (the optical density of each sample indexed to the positive and negative controls on each plate) + a constant. Blue bars indicate samples from individuals without cysts, while grey bars indicate samples from individuals with cysts. The dotted line indicates the optimal threshold cutoff for positive samples indicating antigen presence calculated with the ROC analysis.

Twenty-six of 158 individuals without visible cysts (16.4%) tested positive at least once. This included 14 females and 12 males, of which 6 were subadults and 20 were adults. All but one sample from an individual with a visible cyst fell above the optimal cutoff (Fig 3), indicating that samples from individuals with cysts had generally higher logged index values (IVs) than individuals without cysts. Importantly, 2 individuals without cysts that tested antigen-positive developed observable cysts within 7 months of sampling. One of these individuals had one negative and one positive sample in the 3 months prior to exhibiting an observable cyst, after which all of his samples were positive. The other individual had one positive sample 7 months before exhibiting an observable cyst, after which all of her samples were positive.

To search for evidence of established *T*. *serialis* infection in individuals without visible cysts, we focused on individuals that were sampled at least 5 times during the study period (21 adults, 2 subadults). We found that some individuals without cysts were consistently positive for *T*. *serialis* antigen, others were consistently negative, and still others switched between antigen-positivity and antigen-negativity throughout the study period. Twelve individuals showed no antigen-positive samples, 2 showed a clear majority of positive samples (one with 8/9 positive samples, one with 9/10 positive samples), and 7 individuals had a single positive sample within a sequence of negative samples. The remaining 2 individuals showed an interesting mixture of positive and negative samples: one individual tested positive in 3 consecutive months, and then negative 7 months later. The other displayed a sequence of negative and positive samples within 6 months.

### Predictors of visible *T*. *serialis* cysts and *T*. *serialis* antigen in urine

We investigated the predictors of visible cysts, focusing on age, sex, and the interaction between these two variables. AICc model selection revealed the models with the most support to include age (in years), sex, and an interaction between age and sex as predictor variables, with the model including only age garnering the most support (Table 2). The importance of age and lack of effect of sex were reinforced with the results of full model averaging, which showed that increasing age was the strongest predictor of cysts across all models (Table 3).

**Table 2:** AICc model selection for predictors of *T*. *serialis* cysts in geladas.

Model	Intercept	Years	Male	M:Y	AICc	Δ	Weight
**1.1. Cyst ~ Years**	-5.00	0.19	NA	NA	70.9	0	0.528
**1.2. Cyst ~ Male + Years**	-5.42	0.21	0.56	NA	72.4	1.51	0.248
**1.3. Cyst ~ Male + Years + Male:Years**	-4.79	0.17	-2.23	0.23	72.6	1.75	0.22

The ‘top model set’ presented here includes all models within <2 Δ AICc points of the best model. Predictor coefficient intercepts, AICc values, Δ scores, and weights of each model are given.

**Table 3:** Full model averaged coefficient estimates for the predictors of *T*. *serialis* cysts in geladas (Model 1), and the predictors of antigen-positivity (Models 2 & 3).

Model	Variable	Estimate	SE	z-value	Pr(|>z|)
*1*. Cyst ~ Male * Years	Intercept	-5.06	0.97	5.22	2e-07 *
	Years	0.19	0.06	3.06	<0.01*
	Male	0.35	0.60	0.22	0.83
	M:Y	0.05	0.13	0.39	0.70
*2*. Positive ~ Male * Age + (1| ID)	Intercept	-7.79	1.15	6.77	<2e-16 *
	Age (Adult)	0.06	0.55	0.10	0.92
	Male	NA	NA	NA	NA
*3*. Positive ~ Male * Years + (1| ID)	Age (Adult)	-7.70	1.22	6.34	<2e-16 *
	Male	-0.01	0.07	0.11	0.91

Averaged parameter estimates, adjusted standard errors (SE), z-values, and probability estimates (Pr (>|z|) are presented for all predictor variables in each model. Results are presented for both full model averaging and conditional averaging, and are rounded to the nearest hundredth. Statistical significance is marked by an asterisk (*).

We then investigated the predictors of antigen-positivity in urine samples, again including age, sex, and the interaction between these two variables as predictors. One analysis included age coded categorically, whereas the other included age coded continuously, and both included individual ID as a random intercept to account for repeated sampling from individuals. In the first analysis (categorical age), AICc model selection showed that the model with the most support included only the random intercept (individual ID) and no fixed effects (i.e., age, sex, and the interaction did not appear as predictors of Taenia antigen-positivity in samples, Table 4). A model including age and the random intercept was less supported than the model containing only the random intercept (Table 4). Full model averaging revealed age to be a weaker predictor of antigen-positivity than the random intercept (Table 3). Results were similar for the analysis that used age coded as a continuous variable. The model with the most support included only the random intercept and no fixed effects (Table 4), which was also reflected in the model averaging estimates (Table 3).

**Table 4:** AICc model selection for predictors of *T*. *serialis* antigen-positivity in gelada urine.

Model	Intercept	Years	Age	AICc	Δ	Weight
**Positive ~** ***Categorical Age*** **2.1. (1 | Name)** **2.2 Age + (1 | Name)**	-7.76 -7.88	NA NA	NA 0.21	220.12 222.11	0 1.99	0.73 0.27
***Continuous Age*** **3.1. (1 | Name)** **3.2. Years + (1 | Name)**	-7.76 -7.54	NA -0.03	NA NA	220.12 222.10	0 1.98	0.73 0.27

The ‘top model set’ presented here includes all models within <2 Δ AICc points of the best model. Predictor coefficient intercepts, AICc values, Δ scores, and weights of each model are given. Models 2.1 and 2.2 include age as a categorical value (i.e., adult, subadult), and models 3.1 and 3.2 include age as a continuous variable.

## Discussion

We adapted and evaluated a monoclonal antibody-based sandwich ELISA protocol for the detection of *Taenia* antigen in dried gelada urine, finding that our adaptation was able to detect *Taenia* antigen with high accuracy in geladas infected with *T*. *serialis*. We implemented this assay in a wild gelada population in order to understand basic patterns of *T*. *serialis* infection, providing the first evidence for widespread *T*. *serialis* infection in individuals that do not exhibit external cysts. Our results indicate that *T*. *serialis* infection is more widespread than are visible cysts, with 18% of the sampled population testing positive for *Taenia* antigen where only 4.8% exhibited visible cysts. However, our results demonstrate the occurrence of short-term antigen presence in individuals sampled multiple times, suggesting that individuals may eliminate initial infection with *T*. *serialis* and that a single positive sample may not necessarily indicate an established infection (as do cysts).

### Assay adaptation and evaluation

Positive antigen samples are highly likely to reflect active larval growth (i.e., true infections) and not merely the presence of eggs passing through the gastrointestinal tract, because this assay identifies active infection by detecting glycoproteins produced by taeniid metacestodes and not oncospheres (this also precludes the possibility that positive antigen samples reflect atypical growth of the adult stage of the tapeworm in geladas) [75, 95]. We postulate that individuals without cysts that presented with high log(IV) samples should be considered positive for *Taenia* antigen and are likely to harbor active infections that are not visible as cysts to observers, whether because (1) the infection is young and has not yet had time to develop into a visible cyst; or (2) the infection is advanced but is located deep in the abdominal cavity or somatic tissues and will never become visible. It is highly unlikely that the samples positive for antigen presence are all false positives: based on the false positive rate of 1.79% calculated using the “known negative” infant set (in which 1 out of 57 samples from unweaned infants tested positive), the expected number of false positives is 8.4, and the probability of observing 50 or more false positives in 412 samples is less than p = 10^−25^. These two possibilities–that positive assay results indicate young infections or fully developed internal cysts–are not mutually exclusive.

In support of the interpretation of a positive antigen result as (1) reflecting the presence of young cysts that are not yet observable externally, 2 individuals that tested positive with no external cysts at the time of sample collection developed cysts within a year of sampling. In support of the interpretation of a positive antigen result as (2) reflecting the presence of advanced infections in deep tissue that will never become visible to observers, early necropsies of wild-caught captive geladas revealed fully developed, non-protruding cysts in the abdominal cavities, deep musculature, and viscera [39–41, 79–83]. Thus, positive assay results in the absence of observable cysts may reflect either young infections or advanced infections in undetectable locations.

### Individual infection status

Interestingly, we observed switches in infection status (antigen-positive or antigen-negative) within individuals without cysts (i.e., positive to negative and vice versa). Among 23 well-sampled individuals without cysts (i.e., 5 or more samples), only 2 had a clear majority of antigen positive samples, whereas 12 had no positive samples, 7 had just 1 positive sample, and the remaining 2 flipped from positive to negative during the study period. The observed switches in infection status may reflect either (1) the inability of some larvae to persist; or (2) the ability of hosts to control or eliminate their infections through calcification (although caveats in data certainty must also be considered, such as incorrect individual identification during sample collection). Importantly, the values of samples from these individuals were strikingly different enough (i.e., not close to the cutoff on either side) to make it unlikely that variation in sample quality was behind this pattern. A similar phenomenon was described in humans with *T*. *solium* cysticercosis, with 3.5% of 867 participants exhibiting a single positive sample in between 2 negative samples [96]. The authors postulated that this short-term antigen presence could owe to incomplete parasite formation or to effective host defenses that enable clearance of the parasite.

In geladas, short-term antigen presence may indicate low *T*. *serialis* egg viability or highly effective host immune responses that result in stunted infections or incomplete parasite establishment. Indeed, experimental infection of swine with *T*. *solium* eggs demonstrated low rates of infection establishment even with high infectious doses [97]. Attempts by the host immune system to control infection may not always be successful; for example, one individual tested positive once and negative once in the 3 months before developing an external cyst, after which he consistently tested positive. This may indicate a process in which the host attempted to mount an immune response and was fleetingly able to control the infection before succumbing. Early stages of infection may also release antigens less reliably, which would make early infection difficult to detect. Future work that combines frequent longitudinal urine sampling from known individuals while monitoring for external signs of disease is needed to better understand the frequency and health consequences of transient *T*. *serialis* infections.

### Predictors of *T*. *serialis* infection in urine samples

The higher occurrence of cysts among older individuals is consistent with previous studies of *T*. *serialis* cyst prevalence in geladas [43, 44], whereas the lack of support for a strong relationship between age and antigen-positive samples was unexpected. Together, these results suggest that susceptibility to infection does not vary strongly with age, and that cysts may take years to develop to a stage at which they protrude and are visible to observers. Contrary to our predictions based on the increased female susceptibility observed in other larval taeniid systems [85, 86] or the female-bias in data collection, we found no evidence for a sex bias in either *T*. *serialis* cysts or antigen-positivity in samples. The lack of support for increased susceptibility with age or sex suggests that susceptibility to *T*. *serialis* in geladas may not be hormonally modulated.

Further research is needed to elaborate the physiological and ecological drivers of susceptibility and exposure in this system. Ongoing research is exploring the relationship between co-occurrence of gastrointestinal parasites and *T*. *serialis* infection in geladas, and research is planned to investigate the associations between measurements of stress (fecal glucocorticoid concentrations) and susceptibility to *T*. *serialis* infection and the development of cysts. Future studies should additionally consider other potential drivers of susceptibility and exposure to *T*. *serialis*, such as seasonal changes in *T*. *serialis* egg distribution and gelada ranging patterns and differences in social behavior that affect risk. Articulating the risk factors associated with infection in geladas may inform the understanding of the danger *T*. *serialis* poses to other primates, including humans, as well as the control of infections. If exposure is the central driver of infection, then humans and nonhuman animals that overlap significantly with *T*. *serialis* definitive hosts may be at the highest risk for infection and can thus be targeted for control efforts. While research has shown that *T*. *serialis* cysts substantially increase gelada mortality [45], there is no indication that this infection threatens population-level persistence. Continuous monitoring of *T*. *serialis* and mortality in this population will determine whether future interventions are necessary.

### Limitations and applications

The use of dried urine for larval *Taenia* infection diagnosis provides the substantial benefits of not requiring refrigeration or invasive procedures; thus, it is well suited to the identification of *Taenia* infections in wildlife inhabiting remote areas. However, this approach has one notable drawback: this assay is genus-specific, not species-specific, and will pick up antigens from any *Taenia* species. Thus, other methods must be used for species-level identification. If it is possible to obtain tissue from the cyst of an infected individual (from a dead individual, as in [42], or from leaked cystic material, as in [43]), genetic methods can be used to identify the parasite to the species-level. Non-lethal traps may be employed in studies of smaller species (e.g., lining the trap floor with filter paper for urine collection prior to release), and fecal analysis of carnivore hosts sympatric with the target intermediate host species may also be employed to identify the taeniid species active in a given system.

In future applications of this method, the potential for cross-reactions should be considered. Infection with parasites in the *Trypanosoma* genus may give rise to a cross-reaction on this assay [98], and thus must be taken into account in the interpretation of assay results in *Trypanosoma*-endemic areas. Because geladas inhabit cool, high-altitude habitats that are free of the tsetse flies that carry *Trypanosoma* parasites [99, pers.obs.], and because infection with mechanically transmitted *Trypanosoma* spp. is unlikely in African primates, this cross-reaction was not considered in the interpretation of our results.

In conclusion, the global distribution and flexibility in intermediate host selection of many taeniid species make them critically important to monitor for global human and animal health. The adaptation of a serum protocol for the detection of *Taenia* infections for use with dried urine samples is a useful and pioneering step towards a complete understanding of the dynamics of *Taenia* infection in wildlife. While this assay cannot be used as a stand-alone diagnostic technique, particularly given its genus-wide specificity, it holds great value for studies of infection dynamics in host populations where regular invasive monitoring is impractical and in areas where sample storage prohibits the collection of wet urine samples.

## Supporting information

S1 Text Full adapted protocol for the detection of *Taenia* antigen in dried urine. (DOCX)
