# Supplementary material for: Identifying wildlife reservoirs of neglected taeniid tapeworms: Non-invasive diagnosis of endemic Taenia serialis infection in a wild primate population
Source: PLoS Negl Trop Dis. 2017 Jul 13;11(7):e0005709. doi: 10.1371/journal.pntd.0005709 (PMC5526605; doi:10.1371/journal.pntd.0005709)
Supplement: S1 Text — (DOCX) [file pntd.0005709.s001.docx]

**Adapted protocol for the detection of *Taenia* antigen in dried urine samples in geladas (*Theropithecus gelada*)**

This protocol was adapted from: Department of Animal Health. Detection of viable metacestodes of *Taenia* spp. in human, porcine and bovine serum samples with the use of a monoclonal antibody-based sandwich ELISA. Institute of Tropical Medicine, 2009.

**Materials**

- **ELISA plates**
  - Nunc MaxiSorp® flat-bottom 96 well plate
- **Incubator**
  - VWR 1565 Forced-Air Incubator
- **Shaker**
  - Orbit^TM^ P4 Digital Microplate Shaker
- **Washer**
  - Biotek^TM^ ELx50^TM^ Microplate Strip Washer
- **Reader**
  - Softmax Pro Data Acquisition and Analysis Reader and Software
- **UV Lamp**
  - Spectroline Model ENF-240c (115v, 60 Hz, .20 amps), long-wave UV 365 nm
- **Automatic pipettes**
- **50ml falcon tubes**
- **Plastic basins**
- **Glass bottles**
- **Hole-puncher**

**Buffers and Products**

- Phosphate Buffered Saline (PBS)
  - .01M, pH 7.2
- Tween 20
- Newborn Calf Serum (NBCS)
- Coating Buffer
  - .05M NaHCO_3_ + .05M Na_2_CO_3_ (pH= 9.5)
- Capturing monoclonal antibody
  - B158C11A1 (5µg/mL concentration)
- TMB Substrate
  - Thermo Scientific Pierce 1-Step Ultra TMB-ELISA
- Sulfuric acid
  - H_2_SO_4_
- Detecting monoclonal antibody
  - B60H8A4 (1.25µg/mL concentration)
- Streptavidin-HRP conjugate
- Taenia crassiceps antigen
  - 2.48mg/mL concentration

**Preparation**

1. **Prepare PBS-Tween 20**
   1. Fill graduated glass 1L bottle to 1L mark with .01M PBS
      1. Add magnetic stirbar and place on stir plate at ~3.5rpm (high enough to create a tiny tornado on the top of the liquid)
      2. Add 3mL of Tween to center of PBS
         1. Ensure that Tween is added while the PBS is being stirred, and that Tween does not touch sides of bottle.

3. Let stir for ~15 minutes

1. **Thaw frozen components**
   1. Normal Bovine Calf Serum (NBCS) or Fetal Calf Serum (FBS)
   2. B60H8A4 and B158C11A1 (detecting and capturing antibodies, respectively)

**III. Prepare sensitization buffer:**

- - - Need 9.6mL of sensitization buffer per plate, make 11mL
    - Add 2.6µl B158/1mL of Na_2_CO_3_NaCHO_3_ for a 1.25µg dilution; thus, add 28.6µl B158 to 11mL of Na_2_CO_3_NaCHO_3_

**IV. Prepare blocking buffer (PBS-Tween + 1% NBCS)**

- - Need 113.6mL of BB, make 127mL
    - Blocking step (150µl/well/96 wells=14.4mL)=14.4mL (round to 20mL)
    - Reconstitution step (1mL/sample/80 samples)=80mL (round to 85mL)
    - B60 dilution (100µl/well/96 wells)=9.6mL (round to 11mL)
    - Streptavidin conjugate (100µl/well/96 wells)=9.6mL (round to 11mL)
- Blocking buffer dilution
  - 1µl NBCS : 100µl PBS-Tween
  - 1,270µl NBCS : 127,000µl PBS-Tween
  - 1.27mL NBCS : 127mL PBS-Tween

1. Fill 250mL graduated glass beaker with 127mL PBS-Tween
2. Add 1.27mL NBCS to PBS-Tween.
3. Stir

**V. Prepare B60H8A4 dilution**

- Need 9.6mL of B60 per plate, make 11mL
- If using B60 with 2.2µl/ml concentration, add 24.2µl B60 to 11mL of BB
- If using B60 with 2.0µl/ml concentration, add 22µl B60 to 11mL of BB.
- Vortex

**VI. Prepare Streptavidin conjugate**

- Need 9.6mL of Streptavidin conjugate per plate, make 11mL
- 1µl streptavidin/10ml BB, thus 1.1µl/11ml BB
- In 15 or 50mL Falcon tube, add 1.1µl streptavidin to 11mL of BB.
- Vortex

**VII. Prepare positive titration samples**

- Use *T. serialis* antigen to make 2-fold serial dilutions in 8 known negative samples, vortex.
  - A- 1µg antigen/mL urine
  - B- .5µg antigen/mL urine
  - C- .25µg/mL urine
  - D- .125µg/mL urine
  - E- .0625µg/mL urine
  - F- .03125µg/mL urine
  - G- .015625µg/mL urine
  - H- .0078µg/mL urine

**Protocol**

1. **Dried urine filter paper preparation**
   1. Label each sample tube with associated sample number (side and cap)
   2. In a dark room, remove filter paper sample and observe under UV lamp (long wave, 365nm) for urine stain
   3. Use the hole-puncher to punch four (4) holes from urine-stained sections of filter paper
   4. Transfer four holes to associated sample tube.
2. **Coating Step: coat plate with sensitization buffer (B158 + carbonate bicarbonate buffer)**
   1. Vortex sensitization buffer before use
   2. Pour sensitization buffer into labeled plastic basin
   3. Add 100µl/well using multichannel pipette
   4. Cover plate with sealing film
   5. Place on microplate shaker in incubator
   6. Incubate for 30 minutes at 37°C, while shaking (~750rpm)
3. **Reconstitution Step: reconstitute dry urine samples with blocking buffer**
   1. Pour ~85mL BB into labeled basin
   2. Pipette 1mL BB into each sample tube.
4. **Washing Step: retrieve coated plate from incubator, dump contents into sink, and wash one time with PBS-Tween 20.**
5. **Blocking Step: block wells with 150µl/well blocking buffer (PBS-Tween 20 + 1% NBCS)**
   1. Pour ~15mL of blocking buffer into labeled basin
   2. Add 150µl of BB into each well using multichannel pipette
   3. Cover plate with sealing film
   4. Place on microplate shaker in incubator
   5. Incubate for 15 minutes at 37°C, while shaking (~750rpm)
6. **Sample Loading Step: prepare 80 samples plus four (4) negative controls, two (2) positive controls, and positive titration (8 samples) for loading**
   1. Vortex reconstituted samples
   2. Retrieve blocked plate from incubator
   3. Dump contents into sink, but do not wash
   4. Load 100µl of each reconstituted urine sample into wells according to template
   5. Cover plate with sealing film
   6. Place on microplate shaker in incubator
   7. Incubate for 45 minutes at 37°C, while shaking (~750rpm)
7. **Washing Step: retrieve plate from incubator, wash 5 times with PBS-Tween 20**
8. **Detecting Step: add detecting antibody dilution (B60H8A4 + blocking buffer)**
   1. Vortex B60 dilution before use
   2. Pour 11mL of B60 dilution into labeled basin
   3. Add 100µl of B60 dilution into each well using multichannel pipette
   4. Cover plate with sealing film
   5. Place on microplate shaker in incubator
   6. Incubate for 15 minutes at 37°C, while shaking (~750rpm)
9. **Washing Step: retrieve plate from incubator, wash 5 times with PBS-Tween 20**
10. **Conjugate Step: add Streptavidin-HRP conjugate (Streptavidin HRP conjugate + blocking buffer)**
    1. Vortex Streptavidin-HRP conjugate dilution before use
    2. Pour 11mL of Streptavidin-HRP dilution into labeled basin
    3. Add 100µl of Streptavidin-HRP dilution to each well using multichannel pipette
    4. Cover plate with sealing film
    5. Place on microplate shaker in incubator
    6. Incubate for 15 minutes at 37°C, while shaking (~750rpm)
11. **Washing Step: retrieve plate from incubator, wash 5 times with PBS-Tween 20**
12. **Staining Step: add Tetramethylbenzidine (TMB)**
    1. Pour 11mL of TMB into labeled basin
    2. Add 100µl of TMB into each well using multichannel pipette
    3. Place on shaker and shake at room temperature for two (2) minutes (~600rpm) (?)
13. **Stop Reaction Step: add sulfuric acid (H_2_SO_4_)**
    1. Pour sulfuric acid into labeled basin
    2. Add 100µl of sulfuric acid to each well using multichannel pipette
14. **Reading Step:**
    1. Wipe bottom of plate to ensure clean reading
    2. Ensuring that the orientation of the plate matches the template, load into reader and read at 450nm.
